# Otto: a 4.04 GBq (109 mCi) ^68^Ge/^68^Ga generator, first of its kind - extended quality control and performance evaluation in the clinical production of [^68^Ga]Ga-PSMA-11

**DOI:** 10.1186/s41181-019-0087-y

**Published:** 2020-02-03

**Authors:** Nicole N. Waterhouse, Alejandro Amor-Coarasa, Anastasia Nikolopoulou, John W. Babich

**Affiliations:** 1 Citigroup Biomedical Imaging Center, Weill Cornell Medicine, 516 E 72nd St, New York, NY 10021 USA; 2 Radiochemistry Laboratory, Albert Einstein College of Medicine, Department of Radiology, Montefiore Medical Center, Bronx, NY USA; 3 Division of Radiopharmaceutical Sciences, Department of Radiology, Weill Cornell Medicine, Belfer Research Building, Room 1600, 413 E 69th St, New York, NY 10021 USA; 4 Molecular Imaging Innovations Institute (MI3), Department of Radiology, Weill Cornell Medicine, New York, NY USA; 5 Sandra and Edward Meyer Cancer Center, Weill Cornell Medicine, New York, NY USA

**Keywords:** ^68^Ga/^68^Ga generator, ^68^Ga, Gallium-68, [^68^Ga]Ga-PSMA-11

## Abstract

**Background:**

Here we report on the comprehensive quality control of a 4.04 GBq (109 mCi) generator supplied by itG (Munich, Germany), and used for routine production of [^68^Ga]Ga-PSMA-11 for clinical imaging. The performance of the 4.04 GBq itG ^68^Ge/^68^Ga generator was studied for a year and parameters including elution yield, elution profile, radioactive and stable contaminants were collected. The production yields of a series of 175 [^68^Ga]Ga-PSMA-11 clinical batches are also reported herein.

**Results:**

This first-of-its-kind GMP grade ^68^Ge/^68^Ga generator from itG with a nominal activity of 4.04 GBq (109 mCi) showed a stable ^68^Ga elution profile with elution efficiency averaging 58.3 ± 3.7%. ^68^Ge contaminant in the eluent slightly increased over time but remained 100x lower than those reported for comparable 1.85 GBq (50 mCi) itG generators. Metal impurities were found in concentrations lower than 100 ng/ml (ppb) throughout the study. [^68^Ga]Ga-PSMA-11 was obtained in 89 ± 4% radiochemical yields and > 99% radiochemical and chemical purities.

**Conclusion:**

4.04 GBq (109 mCi) itG ^68^Ge/^68^Ga generator is suitable for routinely produced ^68^Ga tracers used in the clinic. Up to 30% higher amount of final drug product was obtained as compared to the 1.85 GBq (50 mCi) itG generator, and as a result larger number of studies could be performed, while reducing the synthetic burden.

## Key points

QUESTION: Is it possible to scale existing ^68^Ge/^68^Ga generator technology to 3.7 GBq (100 mCi) without affecting performance for clinical use?

PERTINENT FINDINGS: A GMP grade itG ^68^Ge/^68^Ga Generator with a nominal activity of 4.04 GBq (109 mCi) at calibration was studied over a year resulting in unparallel elution reproducibility and affording ^68^Ga activity at an almost stable 58.3 ± 3.7% elution efficiency. A total of 175 clinical productions of [^68^Ga]Ga-PSMA-11 were performed with an 89 ± 4% average radiochemical yield and > 99% radiochemical and chemical purity, producing up to 30% more drug product activity when compared to a typical 1.85 GBq (50 mCi) generator.

IMPLICATIONS FOR PATIENT CARE: This ^68^Ge/^68^Ga generator doubles the initial activity of existing generators accommodating higher patient volumes and resulting a longer shelf life while still performing according to specifications.

## Introduction

**Fig. 1:**
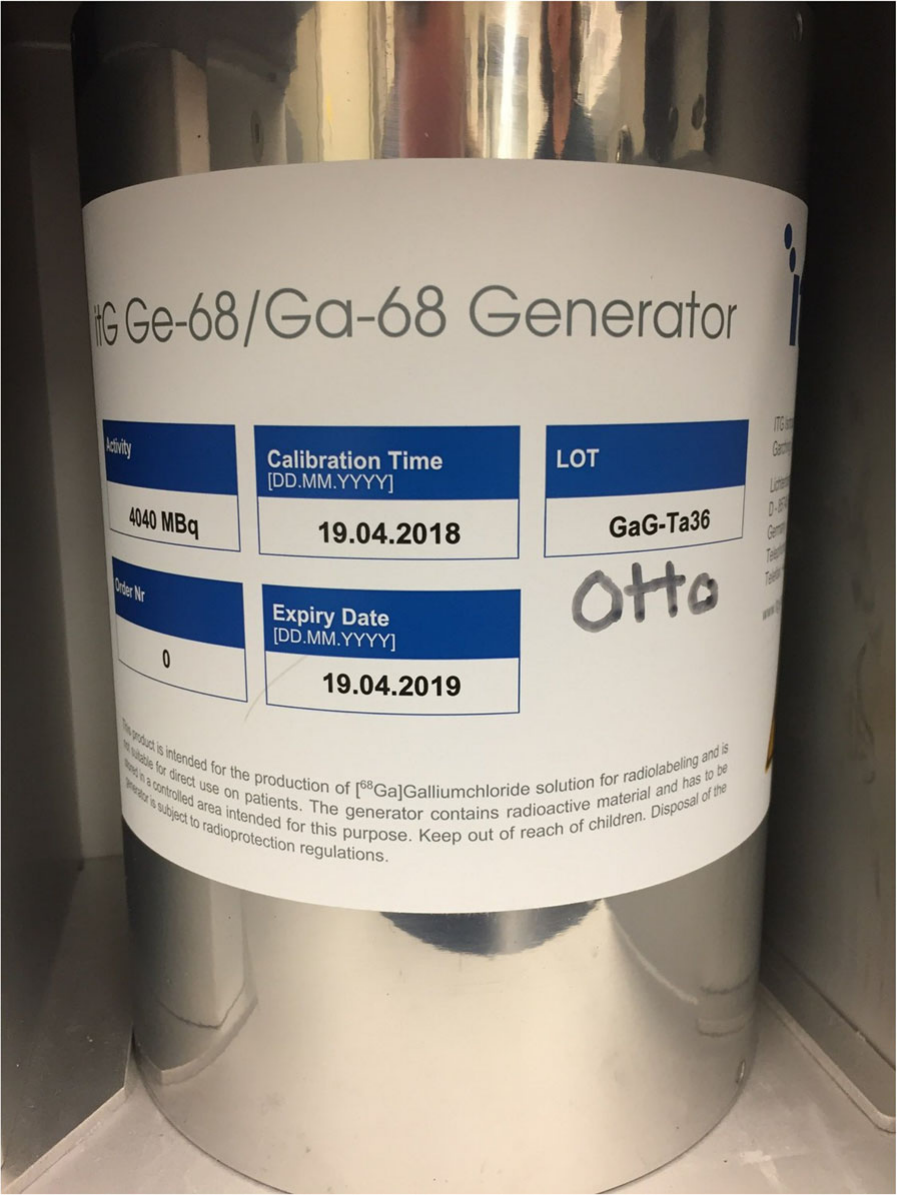
Otto: itG GMP 4.04 Gbq (109 mCi at calibration on 04/19/2018) ^68^Ge/^68^Ga Generator

The value of PSMA-targeted diagnosis and therapy monitoring of prostate cancer by means of PET/CT imaging is undeniable (Hana et al. 2018). While several groups are working on an ^18^F-labeled substitute for PSMA imaging (Kelly et al. 2017; Giesel et al. 2017; Szabo et al. 2015), [^68^Ga]Ga-PSMA-11 (a.k.a. [^68^Ga]Ga-PSMA-HBED-CC or [^68^Ga]Ga-DKFZ-PSMA-11) is the current gold standard (Hana et al. 2018). However, PET/CT imaging with [^68^Ga]Ga-PSMA-11 is becoming a victim of its own success, and the increasing patient volume is calling for either the increase in generator production or the availability of generators containing higher initial activity, or both (Smith et al. 2013). Despite efforts to directly produce Gallium-68 (^68^Ga) in cyclotrons and because of many technical and financial complications (Pandey et al. 2014), currently ^68^Ga can only be reliably produced using a ^68^Ge/^68^Ga generator (Amor-Coarasa et al. 2016, 2017; McElvany et al. 1984; Amor-Coarasa et al. 2018). To date, the commercially available ^68^Ge/^68^Ga generators do not exceed the capacity of 1.85 GBq (50 mCi) (Amor-Coarasa et al. 2016, 2017, 2018; McElvany et al. 1984; Roesch 2013; Greene and Tucker 1961). Here we report a comprehensive quality control of a 4.04 GBq (109 mCi) ^68^Ge/^68^Ga generator produced by Isotopen Technologies Garching GmbH (itG GmbH, Munich, Germany); herein lovingly and appropriately referred to as “Otto” (Fig. 1). We also evaluate its use in the routine clinical production of [^68^Ga]Ga-PSMA-11 in combination with an iQS Fluidic Labeling Module.

## Materials and methods

Otto was received 40 days post calibration from Isotopen Technologies Garching GmbH (itG GmbH, Munich, Germany), containing 4.04 GBq (109.2 mCi on April 19, 2018) of Germanium-68 (^68^Ge). Otto is a metal free, GMP ^68^Ge/^68^Ga generator, based on an Dodecyl-3,4,5-trihydroxybenzoate hydrophobically bounded to an Octadecyl modified silica resin (C-18 resin). All elutions were performed with a syringe pump at a flowrate of 2 ml/min to assure consistency (KD Scientific 100 Legacy pump, USA). Hydrochloric acid (HCl, 37%, 99.999% trace metal grade) used for elution was acquired from Sigma-Aldrich, diluted in 18.2 MΩ MilliQ water (Millipore) to obtain a 0.05 M solution for elution. DKFZ-PSMA-11 (GMP) was acquired from Advanced Biochemical Compounds (ABX, Radeberg, Germany). Sterile GMP labeling kits and fluidic cassettes were acquired from itG.

For labeling, Otto was eluted with 4 ml 0.05 M HCl, making sure an elution had been performed at least 24 h in advance. Generator elutions for quality control purposes were performed on a weekly basis - preferably on Mondays after weekend inactivity - using 6 ml 0.05 M HCl and collecting 6 × 1 ml fractions. Collected fractions were assayed for ^68^Ga activity content in a CRC-15 PET Capintec dose calibrator and left to decay for at least 24 h. All decayed fractions were counted to determine ^68^Ge breakthrough (reported as nominal activity, activity concentration, or as % of the total ^68^Ge activity in the generator at the time of elution) using a Wallace Wizard 3″ 1480 well-counter, and a 4.118 kBq (111.3 nCi; calibrated on 8/7/2017) ^68^Ge NIST traceable source was used for quantification. Fractions from elutions performed on days 41, 77, 111, 200 and 322 post-calibration were randomly selected (a representative sample spread over the year of study) and their ^68^Ga and ^68^Ge elution profiles are presented in the Results section. The same decayed fractions were analyzed by ICP-MS to determine the amounts of stable Cr, Mn, Fe, Co, Ni, Cu, Zn, Ga, Ge, and Al contaminants per elution and per fraction.

As stated before, the generator was eluted at least 24 h in advance of any patient study to eliminate excess ^68^Zn from ^68^Ga decay and radiolysis products. To further test generator’s performance, [^68^Ga]Ga-PSMA-11 was labelled using the itG’s iQS ^68^Ga Fluidic Labeling Module and itG’s ^68^Ga Peptide Radiolabeling kit at 95 °C for 5 min as described previously (Amor-Coarasa et al. 2016). Briefly, 5μg of PSMA-11 were added to 1 ml NaOAc buffer solution included in the kit package. [^68^Ga]Ga-PSMA-11 was purified using a reverse phase C18 Sep-Pak Light (Waters, USA) and filtered for sterilization through a Millipore Cathivex-GV 0.22 μm membrane before undergoing quality control testing. All QC testing was also performed as previously described (Amor-Coarasa et al. 2016), and included bubble point test, pH, sterility, decay, MCA, HPLC and pyrogen testing (Additional file 1: Table S4).

## Results

**Fig. 2:**
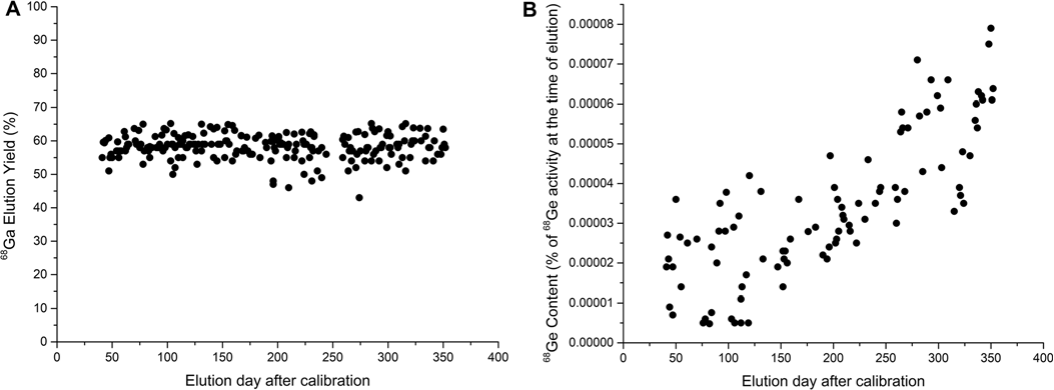
Long term study of Otto. **a**. ^68^Ga elution yield (%) and **b**. ^68^Ge contaminant as % of ^68^Ge activity present in the generator at the time of elution

The ^68^Ge/^68^Ga generator studied herein contained 4040 MBq (109.2 mCi) of ^68^Ge at calibration. This generator was used extensively in our department for almost a year, having undergone 230 elutions for clinical [^68^Ga]Ga-PSMA-11 production and generator quality control as well as > 100 additional elutions for preclinical research (the latter data not included in this study). The average ^68^Ga elution efficiency for this generator was 58.3 ± 3.7% (all reported values are decay corrected). Over the studied period, the elution efficiency remained remarkably consistent, as shown in Fig. 2 (slope ≈0). The maximum elution yield was 65.2% registered at day 103 post-calibration, while the minimum 43.0% was obtained at day 274 (Fig. 2). In contrast to the stable and reproducible ^68^Ga elution yield shown by Otto, the amount of ^68^Ge in the eluting solution increased over time, ranging from 4.8 × 10^− 6^% on day 82 to 7.9 × 10^− 5^% on day 350 post-calibration (and average of 6× increase within the studied period, expressed as % of ^68^Ge present in the generator at the time of elution) (Fig. 2). Despite this increase of ^68^Ge content with time, the amounts always remained under 0.001%, with an average value of (3.4 ± 1.8)·10^− 5^% (Fig. 2).

**Fig. 3:**
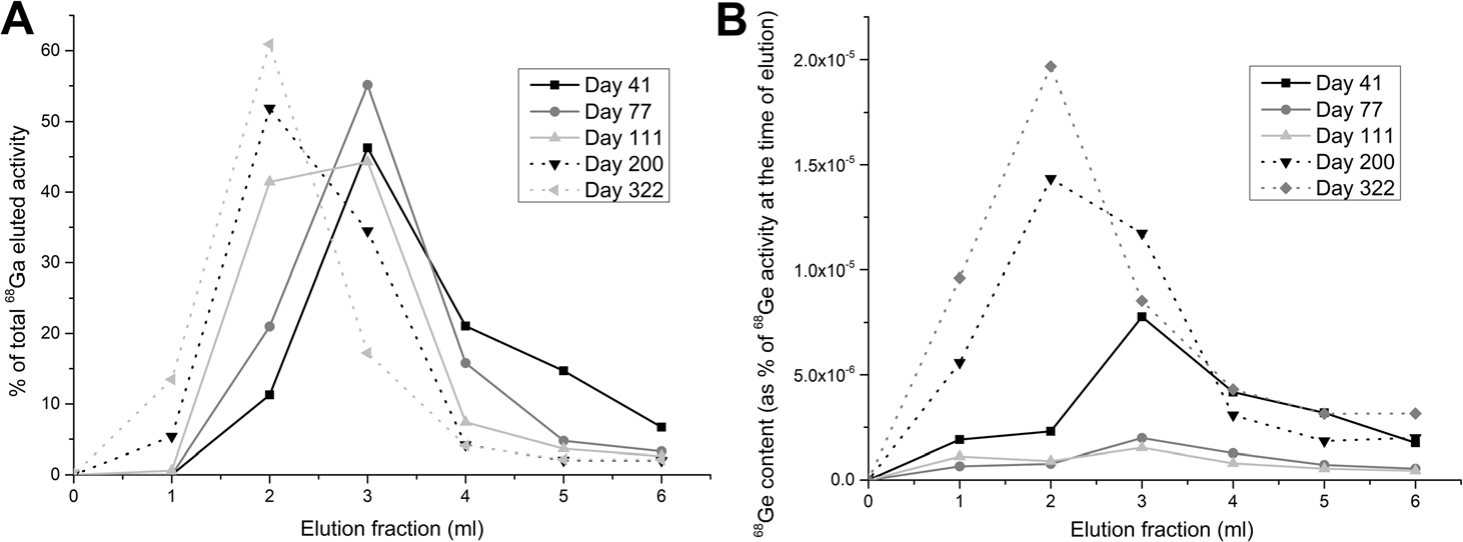
Elution profiles on days 41, 77, 111, 200 and 322 post calibration for **a**: ^68^Ga as percent of total eluted ^68^Ga activity, and **b**: ^68^Ge as percent of the initial ^68^Ge activity present in the generator at the time of elution.

During the first 100 days of use, 69.5±5.6% of the eluted ^68^Ga activity was found in fractions 3 and 4. The elution profile started changing gradually after day 100 with the bulk of the ^68^Ga activity eluted moving towards the elution front; 83.4±3.7% of the activity was found in fractions 2 and 3 (with a reduction to 34.4±13.6% in fractions 3 and 4) (Fig. 3a). The ^68^Ge elution profile also changed in a similar manner, accompanied by an overall increase in the eluted activity (Fig. 3b). Raw data collected is shown in tables in the Additional file 1: Table S1.

**Fig. 4:**
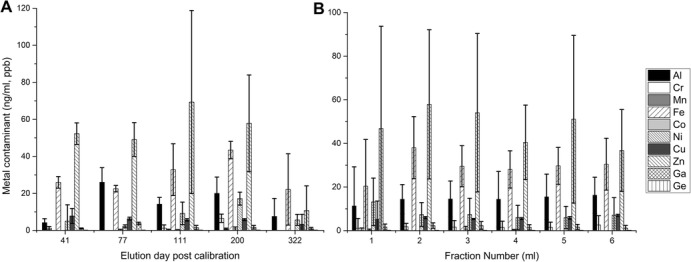
Metal contaminant concentrations (ng/ml, ppb) determined by ICP-MS **a**: concentrations vs the elution number, and **b**: concentration vs elution fraction

The concentrations of metal impurities, such as Cr, Mn, Fe, Co, Ni, Cu, Zn, Ga, Ge, and Al, present in elutions 41, 77, 111, 200, and 322 were extremely low, always under 100 ng/ml (ppb) as shown in Fig. 4. The main impurity present was Zinc, mainly due to ^68^Ga decay. A comprehensive table containing the raw values presented in Fig. 4 is included in the Additional file 1: Table S2.

**Fig. 5:**
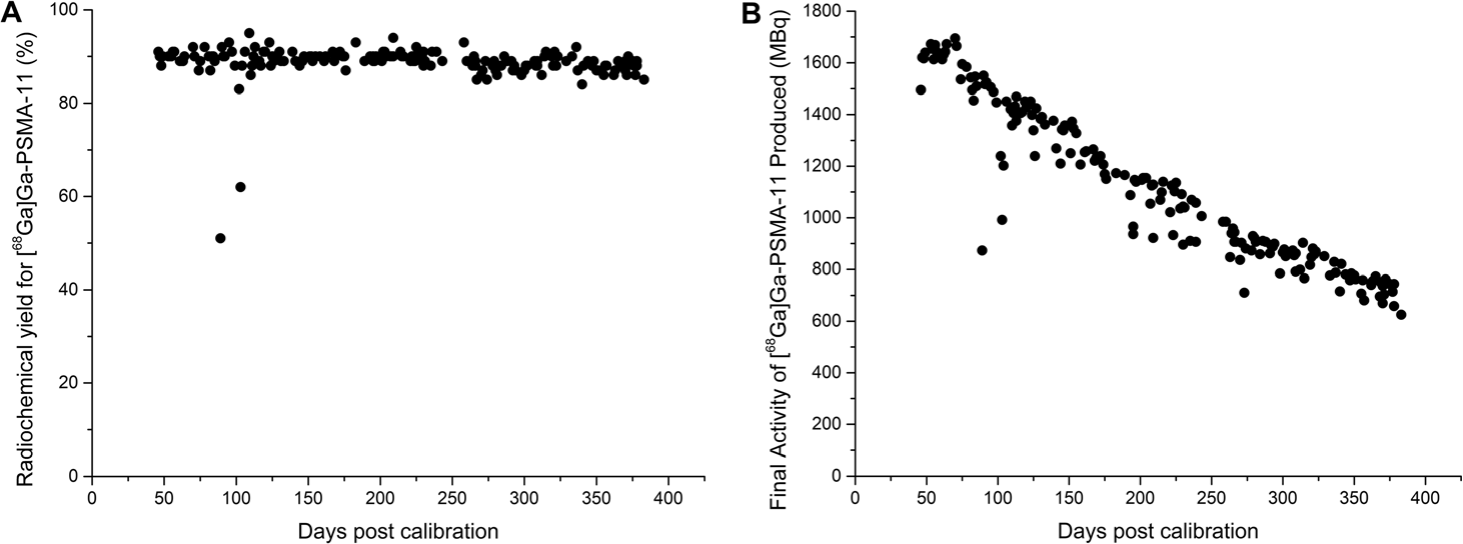
Syntheses of [^68^Ga]Ga-PSMA-11 over the studied period **a**: Radiochemical yields (%) and **b**: Final product activity (MBq)

The average radiochemical yield for 175 [^68^Ga]Ga-PSMA-11 clinical preparations using this generator was 89 ± 4%. The individual radiochemical yields over time (vs. generator age in days post calibration) are presented in Fig. 5. During clinical preparations, the radioactivity found in the waste vial accounted for only 3.4 ± 1.2% of total eluted activity - presumed to be free ionic ^68^Ga – and was not further tested. The C-18 sep-pak lite (used for final drug purification and reformulation) contained 5.5 ± 3.2% of the eluted activity while less than 2% of the activity (presumed [^68^Ga]Ga-PSMA-11, but not extracted for testing) was retained in the 0.22 μm Cathivex filter. The ^68^Ge radionuclidic impurity was not detected in the final drug product (< 50 Bq/ml or 1.5 nCi/ml: detection limit for ^68^Ge in our well-counter) and was found to be at similar levels in the waste vial during synthesis than that in the quality control elutions (Figs. 2 and 3). The radiochemical and chemical purity of the drug product was > 99% for all preparations of [^68^Ga]Ga-PSMA-11, as determined by radio-HPLC.

A table containing the list with the Batch Release Acceptance Criteria for [^68^Ga]Ga-PSMA-11 along with the average results obtained in 175 production syntheses is included in the Additional file 1: Table S4. A table containing the values plotted in Fig. 5 is also included in the Additional file 1: Table S3.

## Discussion

Otto is yet another example of the outstanding performance achieved by modern ^68^Ge/^68^Ga generators. The 100% increase in ^68^Ge activity at calibration when compared to any other reported ^68^Ge/^68^Ga generator, did not led to any measurable increase of ^68^Ge content, radiolysis products or other metal contaminants in the elution. In fact, the purity of the elution of this particular ^68^Ge/^68^Ga generator outperformed any other reported generator in the literature (Amor-Coarasa et al. 2016, 2017, 2018; McElvany et al. 1984; Roesch 2013; Greene and Tucker 1961). The 100% increase in ^68^Ge activity at calibration, resulted in only a 20–30% increase in eluted ^68^Ga activity, due to a decreased elution yield when compared to previously published reports of similar generators from the same manufacturer (Amor-Coarasa et al. 2016, 2017). These elution yields did not appear to change significantly over time, eluting more than 1 TBq (≈27 mCi) of ^68^Ga a year after calibration (Fig. 2). In our clinical setting, this increase in overall eluted activity allowed us to prepare multiple doses of [^68^Ga]Ga-PSMA-11 out of a single clinical production run, thus reducing the overall cost of drug production as well as the labor involved.

The decrease observed in both the ^68^Ge content and the elution yield cannot be explained since we had no part in the production of this generator, but we could speculate that it maybe is the result of incorporating an enlarged column to accommodate the higher initial activity. Interestingly, and contrary to previous reports, the amount of ^68^Ge breakthrough increased along with the generator age, as shown in Fig. 2. Nevertheless, the maximum ^68^Ge breakthrough observed (1.32 kBq or 0.036 μCi), accounts for only 8 × 10^− 5^% of the total ^68^Ge activity present in the generator at the time of elution, which is almost 100-fold lower than the one observed with previous generators at their purest ^68^Ge elution levels.

The elution profiles for both ^68^Ga and the ^68^Ge impurity changed over time. The highest activity concentration was initially found in fraction 3, and later moved to fraction 2. This change is again contrary to what was reported before for smaller generators from the same manufacturer, for which the elution profile was extended with time. While the elution profiles were determined in the 6 ml quality control elution, the elution yields were determined with all elutions (performed with both 6 and 4 ml). Hence, this initially extended profile could have reduced the overall yield measured when eluting with 4 ml 0.05 M HCl for labeling (Fig. 3a). This change in profile can also be partially responsible for the “stable” elution yield observed over time, as well as the minor elution yield variabilities here reported (Fig. 2).

The extended ICP-MS metal contamination study performed here revealed: i) the amounts of Iron contaminant found (main interference in the labeling of [^68^Ga]Ga-PSMA-11) were 10 times lower than the ones reported for previous ^68^Ge/^68^Ga generators from this manufacturer (Amor-Coarasa et al. 2016), ii) the Zinc contaminant was found in similar quantities to previously reported data for previous ^68^Ge/^68^Ga generators - most likely the direct result of accumulation due to ^68^Ga decay and iii) Of all other metals studied, Aluminum concentrations were always found to be the most prominent, however never exceeding 30 ng/ml (ppb). The amounts of metal contaminants did not change significantly during the studied period (*p* > 0.05) and did not showed a marked elution profile (*p* > 0.5, between fractions for all metals), which indicates that fractioning should perhaps be avoided as a purification method for this generator, given that there will not be a reduction in ^68^Ge amounts either (Fig. 3), and valuable ^68^Ga activity will be lost. Another important consideration is that the determination of metal contaminants presented in this report was based exclusively to quality control elution samples collected without the 24 h pre-elution that routinely precedes the clinical production runs of [^68^Ga]Ga-PSMA-11. Therefore, the concentrations reported herein for metal contaminants represent the “worst case scenario” and are estimated to be significantly lower in production elutions.

[^68^Ga]Ga-PSMA-11 syntheses were reproducibly performed with activity eluted from the 4.04 GBq (109 mCi) ^68^Ge/^68^Ga Generator and with an average radiochemical yield of 89 ± 4%. A few lower yield outliers could most likely be linked to operator manipulation errors. As stated before, the ^68^Ge breakthrough in the final drug product was found < 50 Bq/ml (< 5·10^− 6^% of ^68^Ge activity in the generator) at all instances, which is > 200 times below the acceptance criteria of 0.001% for [^68^Ga]Ga-PSMA-11. The waste vial from [^68^Ga]Ga-PSMA-11 production was found to contain the bulk of the ^68^Ge breakthrough from the elution. No radio or UV impurities were noticed in any of the ^68^GaPSMA chromatograms, and all batches showed > 99% radiochemical and chemical purity. The pure and reliable ^68^Ga produced by Otto resulted in a year of reproducible drug production for clinical use. Although typically the manufacturer specified shelf life of ^68^Ge/^68^Ga generators is set to 1 year due to the decrease of ^68^Ga elution yield and the parallel increase in ^68^Ge breakthrough (Amor-Coarasa et al. 2017), this type of ^68^Ge/^68^Ga Generators (Containing approximately 3.7 GBq or 100 mCi, Otto-like) could easily surpass it while still performing according to specifications.

## Conclusion

Otto, the first-of-its-kind GMP grade itG ^68^Ge/^68^Ga Generator with a nominal activity of 4.04 GBq (109 mCi) at calibration, was studied over a year. Otto’s performance showed unparallel reproducibility over the studied period and afforded ^68^Ga activity at an almost stable 58.3 ± 3.7% elution efficiency. Although amounts of ^68^Ge in the elution slightly increased over time, they always remained approximately 100-fold lower than previously reported for generators with lower ^68^Ge load (Amor-Coarasa et al. 2016, 2017, 2018; McElvany et al. 1984; Roesch 2013; Greene and Tucker 1961). Also, the amounts of other metal impurities were lower than the ones measured in previous reports (Amor-Coarasa et al. 2016, 2017, 2018; McElvany et al. 1984; Roesch 2013; Greene and Tucker 1961). A total of 175 clinical productions of [^68^Ga]Ga-PSMA-11 were performed with an 89 ± 4% average radiochemical yield and > 99% radiochemical and chemical purity. Up to 30% more drug product activity was obtained when compared to a typical 1.85 GBq (50 mCi) generator, accommodating higher patient volumes.

## Supplementary information

**Additional file 1: Table S1.** Data compilation: ^68^Ga elution yields and ^68^Ge contents. **Table S2.** Results from ICP-MS for metal contamination. **Table S3.** Compilation of [^68^Ga]Ga-PSMA-11 Syntheses. **Table S4.** Drug product release criteria for [^68^Ga]Ga-PSMA-11. **Figure S5.** Typical QC Chromatogram for [^68^Ga]Ga-PSMA-11.

## Data Availability

All data generated or analysed during this study are included in this published article and its Additional file.
