# Supplementary material for: Otto: a 4.04 GBq (109 mCi) 68Ge/68Ga generator, first of its kind - extended quality control and performance evaluation in the clinical production of [68Ga]Ga-PSMA-11
Source: EJNMMI Radiopharm Chem. 2020 Feb 3;5:5. doi: 10.1186/s41181-019-0087-y (PMC6997319; doi:10.1186/s41181-019-0087-y)

Supplementary Materials

Table SM1. Data compilation: ^68^Ga elution yields and ^68^Ge contents.

| Date | Days post Calibration | ^68^Ga Elution Yield (%) | % ^68^Ge in the elution |
| --- | --- | --- | --- |
| 5/29/2018 | 41 | 55.0 | 0.000019 |
| 5/30/2018 | 42 | 59.6 | 0.000027 |
| 5/31/2018 | 43 | 59.4 | 0.000021 |
| 6/1/2018 | 44 | 60.1 | 0.000009 |
| 6/4/2018 | 47 | 60.9 | 0.000007 |
| 6/4/2018 | 47 | 51.0 | 0.000019 |
| 6/5/2018 | 48 | 55.0 |  |
| 6/6/2018 | 49 | 56.0 |  |
| 6/7/2018 | 50 | 55.0 | 0.000036 |
| 6/11/2018 | 54 | 57.0 | 0.0000265 |
| 6/12/2018 | 55 | 57.0 | 0.000014 |
| 6/12/2018 | 55 | 59.7 |  |
| 6/13/2018 | 56 | 55.0 |  |
| 6/14/2018 | 57 | 57.0 |  |
| 6/15/2018 | 58 | 57.0 |  |
| 6/18/2018 | 61 | 62.8 | 0.000025 |
| 6/19/2018 | 62 | 57.0 |  |
| 6/19/2018 | 62 | 61.2 |  |
| 6/20/2018 | 63 | 58.0 |  |
| 6/21/2018 | 64 | 59.0 |  |
| 6/22/2018 | 65 | 59.0 |  |
| 6/27/2018 | 70 | 63.7 | 0.000026 |
| 6/28/2018 | 71 | 60.0 |  |
| 6/29/2018 | 72 | 59.0 |  |
| 7/1/2018 | 74 | 63.1 |  |
| 7/2/2018 | 75 | 58.0 |  |
| 7/3/2018 | 76 | 59.0 | 0.000005 |
| 7/5/2018 | 78 | 53.0 | 0.000006 |
| 7/5/2018 | 78 | 65.0 |  |
| 7/6/2018 | 79 | 57.0 |  |
| 7/9/2018 | 82 | 58.0 | 0.0000048 |
| 7/10/2018 | 83 | 57.0 |  |
| 7/11/2018 | 84 | 55.0 | 0.000024 |
| 7/11/2018 | 84 | 58.3 | 0.0000076 |
| 7/12/2018 | 85 | 58.0 |  |
| 7/13/2018 | 86 | 58.0 |  |
| 7/16/2018 | 89 | 61.3 | 0.00002 |
| 7/17/2018 | 90 | 58.0 |  |
| 7/18/2018 | 91 | 58.0 | 0.000028 |
| 7/19/2018 | 92 | 58.0 | 0.000035 |
| 7/20/2018 | 93 | 59.0 |  |
| 7/22/2018 | 95 | 61.7 |  |
| 7/23/2018 | 96 | 57.0 |  |
| 7/24/2018 | 97 | 63.7 | 0.000028 |
| 7/25/2018 | 98 | 58.0 | 0.0000378 |
| 7/26/2018 | 99 | 63.3 |  |
| 7/27/2018 | 100 | 59.0 |  |
| 7/30/2018 | 103 | 54.0 | 0.000006 |
| 7/30/2018 | 103 | 65.2 |  |
| 7/31/2018 | 104 | 59.0 |  |
| 8/1/2018 | 105 | 59.0 | 0.000029 |
| 8/1/2018 | 105 | 50.0 |  |
| 8/2/2018 | 106 | 62.2 | 0.000005 |
| 8/3/2018 | 107 | 58.0 |  |
| 8/3/2018 | 107 | 52.0 |  |
| 8/6/2018 | 110 | 55.0 | 0.0000318 |
| 8/6/2018 | 110 | 61.0 |  |
| 8/7/2018 | 111 | 59.0 |  |
| 8/8/2018 | 112 | 59.0 | 0.000005 |
| 8/8/2018 | 112 | 60.3 | 0.000011 |
| 8/9/2018 | 113 | 59.0 | 0.000014 |
| 8/10/2018 | 114 | 60.0 |  |
| 8/10/2018 | 114 | 55.0 |  |
| 8/12/2018 | 116 | 61.6 |  |
| 8/13/2018 | 117 | 58.0 | 0.000017 |
| 8/14/2018 | 118 | 58.0 |  |
| 8/15/2018 | 119 | 61.8 | 0.000005 |
| 8/16/2018 | 120 | 59.0 | 0.000042 |
| 8/17/2018 | 121 | 59.0 |  |
| 8/19/2018 | 123 | 61.3 |  |
| 8/20/2018 | 124 | 59.0 |  |
| 8/21/2018 | 125 | 59.0 |  |
| 8/22/2018 | 126 | 57.0 |  |
| 8/23/2018 | 127 | 53.0 |  |
| 8/24/2018 | 128 | 59.0 |  |
| 8/27/2018 | 131 | 57.0 | 0.000038 |
| 8/27/2018 | 131 | 65.0 |  |
| 8/28/2018 | 132 | 59.0 |  |
| 8/29/2018 | 133 | 62.3 | 0.000021 |
| 8/30/2018 | 134 | 59.0 |  |
| 9/4/2018 | 139 | 64.3 |  |
| 9/5/2018 | 140 | 59.0 |  |
| 9/7/2018 | 142 | 55.0 |  |
| 9/7/2018 | 142 | 63.6 |  |
| 9/10/2018 | 145 | 54.0 |  |
| 9/10/2018 | 145 | 64.1 |  |
| 9/11/2018 | 146 | 59.0 |  |
| 9/12/2018 | 147 | 59.0 | 0.000019 |
| 9/13/2018 | 148 | 59.0 |  |
| 9/17/2018 | 152 | 55.0 | 0.000014 |
| 9/17/2018 | 152 | 63.0 | 0.000023 |
| 9/18/2018 | 153 | 60.0 | 0.000021 |
| 9/19/2018 | 154 | 60.0 | 0.000023 |
| 9/20/2018 | 155 | 64.9 |  |
| 9/21/2018 | 156 | 59.0 | 0.00002 |
| 9/24/2018 | 159 | 54.0 | 0.000026 |
| 9/24/2018 | 159 | 64.6 |  |
| 9/26/2018 | 161 | 63.1 |  |
| 9/27/2018 | 162 | 56.0 |  |
| 9/28/2018 | 163 | 57.0 |  |
| 10/2/2018 | 167 | 61.5 | 0.000036 |
| 10/2/2018 | 167 | 61.6 |  |
| 10/3/2018 | 168 | 57.0 |  |
| 10/4/2018 | 169 | 57.0 |  |
| 10/5/2018 | 170 | 58.0 |  |
| 10/7/2018 | 172 | 61.1 |  |
| 10/8/2018 | 173 | 57.0 |  |
| 10/10/2018 | 175 | 56.0 |  |
| 10/11/2018 | 176 | 56.0 | 0.0000279 |
| 10/12/2018 | 177 | 56.0 |  |
| 10/15/2018 | 180 | 60.8 |  |
| 10/18/2018 | 183 | 61.7 | 0.000029 |
| 10/19/2018 | 184 | 55.0 |  |
| 10/24/2018 | 189 | 59.5 |  |
| 10/25/2018 | 190 | 58.0 | 0.000022 |
| 10/29/2018 | 194 | 54.0 | 0.000021 |
| 10/29/2018 | 194 | 59.9 |  |
| 10/30/2018 | 195 | 61.5 |  |
| 10/31/2018 | 196 | 47.0 | 0.000024 |
| 10/31/2018 | 196 | 48.0 |  |
| 11/1/2018 | 197 | 58.0 | 0.000047 |
| 11/1/2018 | 197 | 60.1 |  |
| 11/2/2018 | 198 | 57.0 |  |
| 11/5/2018 | 201 | 61.3 | 0.000039 |
| 11/5/2018 | 201 | 61.1 |  |
| 11/6/2018 | 202 | 59.0 | 0.000025 |
| 11/7/2018 | 203 | 59.0 | 0.000026 |
| 11/7/2018 | 203 | 59.4 |  |
| 11/8/2018 | 204 | 59.0 | 0.000036 |
| 11/9/2018 | 205 | 59.0 | 0.000028 |
| 11/12/2018 | 208 | 55.0 | 0.000034 |
| 11/12/2018 | 208 | 62.5 |  |
| 11/13/2018 | 209 | 58.0 | 0.000032 |
| 11/14/2018 | 210 | 59.0 | 0.000031 |
| 11/14/2018 | 210 | 46.0 |  |
| 11/19/2018 | 215 | 55.0 | 0.0000295 |
| 11/19/2018 | 215 | 62.0 |  |
| 11/20/2018 | 216 | 57.0 | 0.000028 |
| 11/21/2018 | 217 | 59.0 |  |
| 11/26/2018 | 222 | 54.0 | 0.000025 |
| 11/26/2018 | 222 | 62.6 |  |
| 11/27/2018 | 223 | 60.0 |  |
| 11/28/2018 | 224 | 59.0 | 0.000035 |
| 11/28/2018 | 224 | 50.0 |  |
| 11/29/2018 | 225 | 59.0 |  |
| 11/30/2018 | 226 | 59.0 |  |
| 12/3/2018 | 229 | 55.0 |  |
| 12/3/2018 | 229 | 62.2 |  |
| 12/4/2018 | 230 | 58.0 | 0.000031 |
| 12/4/2018 | 230 | 62.7 |  |
| 12/5/2018 | 231 | 58.0 |  |
| 12/5/2018 | 231 | 48.0 |  |
| 12/6/2018 | 232 | 57.0 |  |
| 12/7/2018 | 233 | 61.8 | 0.000046 |
| 12/7/2018 | 233 | 61.3 |  |
| 12/10/2018 | 236 | 51.0 |  |
| 12/11/2018 | 237 | 57.0 |  |
| 12/14/2018 | 240 | 58.0 | 0.000035 |
| 12/14/2018 | 240 | 49.0 |  |
| 12/18/2018 | 244 | 56.0 | 0.000038 |
| 1/2/2019 | 259 | 55.0 | 0.000039 |
| 1/2/2019 | 259 | 60.4 | 0.000039 |
| 1/3/2019 | 260 | 61.4 | 0.00003 |
| 1/4/2019 | 261 | 59.0 | 0.000036 |
| 1/7/2019 | 264 | 51.0 | 0.000053 |
| 1/7/2019 | 264 | 61.0 |  |
| 1/8/2019 | 265 | 57.0 | 0.000058 |
| 1/9/2019 | 266 | 58.0 | 0.000054 |
| 1/10/2019 | 267 | 57.0 |  |
| 1/10/2019 | 267 | 54.0 |  |
| 1/11/2019 | 268 | 57.0 | 0.000038 |
| 1/14/2019 | 271 | 52.0 | 0.000054 |
| 1/14/2019 | 271 | 62.7 |  |
| 1/15/2019 | 272 | 56.0 |  |
| 1/16/2019 | 273 | 62.8 |  |
| 1/17/2019 | 274 | 43.0 |  |
| 1/18/2019 | 275 | 57.0 |  |
| 1/22/2019 | 279 | 54.0 |  |
| 1/22/2019 | 279 | 61.9 |  |
| 1/23/2019 | 280 | 57.0 | 0.000071 |
| 1/24/2019 | 281 | 58.0 |  |
| 1/25/2019 | 282 | 58.0 | 0.000057 |
| 1/28/2019 | 285 | 54.0 | 0.000043 |
| 1/28/2019 | 285 | 65.2 |  |
| 1/29/2019 | 286 | 63.8 |  |
| 1/30/2019 | 287 | 56.0 |  |
| 1/31/2019 | 288 | 62.8 |  |
| 2/1/2019 | 289 | 57.0 | 0.000058 |
| 2/4/2019 | 292 | 54.0 |  |
| 2/4/2019 | 292 | 63.6 |  |
| 2/5/2019 | 293 | 58.0 | 0.000066 |
| 2/6/2019 | 294 | 58.0 |  |
| 2/7/2019 | 295 | 59.0 |  |
| 2/11/2019 | 299 | 52.0 | 0.000062 |
| 2/11/2019 | 299 | 61.6 |  |
| 2/12/2019 | 300 | 62.8 |  |
| 2/13/2019 | 301 | 58.0 |  |
| 2/14/2019 | 302 | 58.0 | 0.000059 |
| 2/14/2019 | 302 | 61.5 | 0.000044 |
| 2/15/2019 | 303 | 56.0 |  |
| 2/20/2019 | 308 | 59.0 |  |
| 2/21/2019 | 309 | 58.0 | 0.000066 |
| 2/22/2019 | 310 | 59.0 |  |
| 2/22/2019 | 310 | 53.0 |  |
| 2/25/2019 | 313 | 55.0 |  |
| 2/25/2019 | 313 | 64.3 |  |
| 2/27/2019 | 315 | 60.0 | 0.000033 |
| 2/28/2019 | 316 | 51.0 |  |
| 2/28/2019 | 316 | 65.2 |  |
| 3/4/2019 | 320 | 55.0 | 0.000039 |
| 3/5/2019 | 321 | 59.0 | 0.000037 |
| 3/6/2019 | 322 | 60.0 |  |
| 3/7/2019 | 323 | 60.0 | 0.000048 |
| 3/7/2019 | 323 | 63.9 |  |
| 3/8/2019 | 324 | 60.0 | 0.000035 |
| 3/13/2019 | 329 | 63.8 |  |
| 3/14/2019 | 330 | 60.0 | 0.000047 |
| 3/18/2019 | 334 | 54.0 |  |
| 3/18/2019 | 334 | 61.4 |  |

Table SM2. Results from ICP-MS for metal contamination.

| Metal contaminant concentration per elution number in ng/ml (ppb) | | | | | |
| --- | --- | --- | --- | --- | --- |
| Elution # | Al | Cr | Mn | Fe | Co |
| 41 | 4.17±2.15 | 1.20±0.90 | 0.00±0.00 | 25.93±3.14 | 0.00±0.00 |
| 77 | 25.99±7.91 | 0.00±0.00 | 0.00±0.00 | 22.54±1.73 | 0.13±0.47 |
| 111 | 14.17±3.64 | 1.24±1.74 | 0.19±0.42 | 32.82±13.99 | 0.11±0.28 |
| 200 | 20.01±8.81 | 6.55±2.26 | 0.74±0.50 | 43.41±4.69 | 0.86±0.98 |
| 322 | 7.55±9.71 | 0.00±0.00 | 0.00±0.00 | 22.15±19.27 | 0.00±0.00 |
| ALL | **14.38±8.91** | **1.80±2.73** | **0.19±0.32** | **29.37±8.94** | **0.22±0.36** |
| Elution # | **Ni** | **Cu** | **Zn** | **Ga** | **Ge** |
| 41 | 5.00±8.83 | 7.80±4.06 | 52.26±5.83 | 1.03±0.30 | 0.06±0.01 |
| 77 | 2.18±0.82 | 6.35±0.77 | 49.10±9.16 | 3.75±0.67 | 0.06±0.01 |
| 111 | 9.18±6.10 | 5.68±0.66 | 69.35±49.43 | 1.49±1.21 | 0.06±0.01 |
| 200 | 17.15±3.50 | 5.83±0.50 | 57.81±26.14 | 1.69±0.99 | 0.07±0.01 |
| 322 | 5.67±2.98 | 3.35±5.34 | 10.71±13.35 | 0.92±0.68 | 0.03±0.06 |
| ALL | **7.84±5.77** | **5.80±1.61** | **47.85±22.15** | **1.77±1.15** | **0.06±0.02** |
| Metal contaminant concentration per elution fraction in ng/ml (ppb) | | | | | |
| Fraction | **Al** | **Cr** | **Mn** | **Fe** | **Co** |
| 1 | 11.30±17.94 | 1.12±4.46 | 0.11±1.11 | 20.42±21.38 | 0.25±0.45 |
| 2 | 14.38±6.70 | 1.79±1.53 | 0.00±0.00 | 38.04±14.22 | 0.00±0.00 |
| 3 | 14.50±8.26 | 1.61±3.01 | 0.00±0.00 | 29.53±9.22 | 0.70±1.26 |
| 4 | 14.38±12.74 | 1.43±2.88 | 0.00±0.00 | 28.05±8.53 | 0.18±0.36 |
| 5 | 15.46±10.44 | 1.49±2.39 | 0.00±0.00 | 29.70±8.46 | 0.00±0.00 |
| 6 | 16.24±8.24 | 2.59±4.27 | 0.00±0.00 | 30.46±11.88 | 0.00±0.00 |
| ALL | **14.38±16.82** | **1.80±5.01** | **0.19±0.43** | **29.37±56.28** | **0.22±2.73** |
| Fraction | **Ni** | **Cu** | **Zn** | **Ga** | **Ge** |
| 1 | 13.17±10.92 | 5.20±8.34 | 46.82±46.90 | 1.65±1.36 | 0.03±0.07 |
| 2 | 7.37±5.48 | 5.97±0.47 | 57.90±31.21 | 2.29±1.26 | 0.06±0.01 |
| 3 | 7.43±7.39 | 5.33±0.25 | 54.05±36.32 | 2.35±1.80 | 0.06±0.01 |
| 4 | 6.00±5.58 | 5.50±0.44 | 40.48±17.08 | 1.51±1.20 | 0.05±0.02 |
| 5 | 5.97±5.13 | 5.83±0.63 | 51.07±38.44 | 1.65±1.20 | 0.06±0.01 |
| 6 | 7.07±8.04 | 6.99±0.51 | 36.76±18.76 | 1.20±1.17 | 0.06±0.01 |
| ALL | **7.84±26.92** | **5.80±6.49** | **47.85±81.02** | **1.77±4.54** | **0.06±0.12** |

Table SM3. Compilation of [^68^Ga]Ga-PSMA-11 Syntheses.

| Synthesis # | Date | Day Post-calibration | Radiochemical Yield | Final Product (MBq) |
| --- | --- | --- | --- | --- |
| 1 | 6/4/2018 | 46 | 91% | 1494.8 |
| 2 | 6/5/2018 | 47 | 90% | 1620.6 |
| 3 | 6/6/2018 | 48 | 88% | 1616.9 |
| 4 | 6/7/2018 | 49 | 90% | 1639.1 |
| 5 | 6/11/2018 | 53 | 90% | 1672.4 |
| 6 | 6/12/2018 | 54 | 90% | 1653.9 |
| 7 | 6/13/2018 | 55 | 90% | 1613.2 |
| 8 | 6/14/2018 | 56 | 91% | 1668.7 |
| 9 | 6/15/2018 | 57 | 91% | 1642.8 |
| 10 | 6/19/2018 | 61 | 89% | 1613.2 |
| 11 | 6/20/2018 | 62 | 89% | 1631.7 |
| 12 | 6/21/2018 | 63 | 89% | 1642.8 |
| 13 | 6/22/2018 | 64 | 90% | 1672.4 |
| 14 | 6/28/2018 | 70 | 92% | 1694.6 |
| 15 | 6/29/2018 | 71 | 90% | 1665 |
| 16 | 7/2/2018 | 74 | 87% | 1535.5 |
| 17 | 7/3/2018 | 75 | 89% | 1594.7 |
| 18 | 7/6/2018 | 78 | 92% | 1583.6 |
| 19 | 7/9/2018 | 81 | 90% | 1542.9 |
| 20 | 7/10/2018 | 82 | 87% | 1494.8 |
| 21 | 7/11/2018 | 83 | 90% | 1454.1 |
| 22 | 7/12/2018 | 84 | 90% | 1546.6 |
| 23 | 7/13/2018 | 85 | 89% | 1509.6 |
| 24 | 7/17/2018 | 89 | 51% | 873.2 |
| 25 | 7/18/2018 | 90 | 92% | 1550.3 |
| 26 | 7/19/2018 | 91 | 90% | 1517 |
| 27 | 7/20/2018 | 92 | 90% | 1524.4 |
| 28 | 7/23/2018 | 95 | 93% | 1505.9 |
| 29 | 7/25/2018 | 97 | 91% | 1487.4 |
| 30 | 7/27/2018 | 99 | 88% | 1446.7 |
| 31 | 7/30/2018 | 102 | 83% | 1239.5 |
| 32 | 7/31/2018 | 103 | 62% | 991.6 |
| 33 | 8/1/2018 | 104 | 88% | 1202.5 |
| 34 | 8/3/2018 | 106 | 91% | 1450.4 |
| 35 | 8/6/2018 | 109 | 95% | 1420.8 |
| 36 | 8/7/2018 | 110 | 86% | 1357.9 |
| 37 | 8/8/2018 | 111 | 88% | 1406 |
| 38 | 8/9/2018 | 112 | 90% | 1431.9 |
| 39 | 8/10/2018 | 113 | 92% | 1468.9 |
| 40 | 8/10/2018 | 113 | 92% | 1376.4 |
| 41 | 8/13/2018 | 116 | 89% | 1406 |
| 42 | 8/14/2018 | 117 | 88% | 1409.7 |
| 43 | 8/16/2018 | 119 | 91% | 1450.4 |
| 44 | 8/17/2018 | 120 | 91% | 1428.2 |
| 45 | 8/20/2018 | 123 | 93% | 1450.4 |
| 46 | 8/21/2018 | 124 | 88% | 1398.6 |
| 47 | 8/22/2018 | 125 | 90% | 1339.4 |
| 48 | 8/23/2018 | 126 | 89% | 1239.5 |
| 49 | 8/24/2018 | 127 | 90% | 1424.5 |
| 50 | 8/27/2018 | 130 | 91% | 1383.8 |
| 51 | 8/28/2018 | 131 | 89% | 1391.2 |
| 52 | 8/30/2018 | 133 | 89% | 1361.6 |
| 53 | 9/5/2018 | 139 | 91% | 1376.4 |
| 54 | 9/7/2018 | 141 | 89% | 1269.1 |
| 55 | 9/10/2018 | 144 | 88% | 1209.9 |
| 56 | 9/11/2018 | 145 | 89% | 1343.1 |
| 57 | 9/12/2018 | 146 | 89% | 1339.4 |
| 58 | 9/13/2018 | 147 | 90% | 1357.9 |
| 59 | 9/17/2018 | 151 | 90% | 1250.6 |
| 60 | 9/18/2018 | 152 | 90% | 1372.7 |
| 61 | 9/19/2018 | 153 | 89% | 1346.8 |
| 62 | 9/21/2018 | 155 | 90% | 1328.3 |
| 63 | 9/24/2018 | 158 | 90% | 1206.2 |
| 64 | 9/27/2018 | 161 | 89% | 1254.3 |
| 65 | 9/28/2018 | 162 | 90% | 1258 |
| 66 | 10/3/2018 | 167 | 91% | 1265.4 |
| 67 | 10/4/2018 | 168 | 89% | 1221 |
| 68 | 10/5/2018 | 169 | 90% | 1232.1 |
| 69 | 10/8/2018 | 172 | 91% | 1239.5 |
| 70 | 10/10/2018 | 174 | 90% | 1206.2 |
| 71 | 10/11/2018 | 175 | 90% | 1169.2 |
| 72 | 10/12/2018 | 176 | 87% | 1150.7 |
| 73 | 10/19/2018 | 183 | 93% | 1172.9 |
| 74 | 10/25/2018 | 189 | 89% | 1165.5 |
| 75 | 10/29/2018 | 193 | 90% | 1087.8 |
| 76 | 10/31/2018 | 195 | 89% | 936.1 |
| 77 | 10/31/2018 | 195 | 90% | 965.7 |
| 78 | 11/1/2018 | 196 | 89% | 1147 |
| 79 | 11/2/2018 | 197 | 90% | 1139.6 |
| 80 | 11/6/2018 | 201 | 89% | 1147 |
| 81 | 11/7/2018 | 202 | 91% | 1154.4 |
| 82 | 11/8/2018 | 203 | 91% | 1154.4 |
| 83 | 11/9/2018 | 204 | 90% | 1154.4 |
| 84 | 11/12/2018 | 207 | 90% | 1054.5 |
| 85 | 11/13/2018 | 208 | 90% | 1124.8 |
| 86 | 11/14/2018 | 209 | 90% | 1128.5 |
| 87 | 11/14/2018 | 209 | 94% | 921.3 |
| 88 | 11/19/2018 | 214 | 91% | 1069.3 |
| 89 | 11/20/2018 | 215 | 90% | 1098.9 |
| 90 | 11/21/2018 | 216 | 90% | 1139.6 |
| 91 | 11/26/2018 | 221 | 89% | 1021.2 |
| 92 | 11/27/2018 | 222 | 90% | 1124.8 |
| 93 | 11/28/2018 | 223 | 90% | 1121.1 |
| 94 | 11/28/2018 | 223 | 89% | 932.4 |
| 95 | 11/29/2018 | 224 | 89% | 1102.6 |
| 96 | 11/30/2018 | 225 | 92% | 1135.9 |
| 97 | 12/3/2018 | 228 | 91% | 1036 |
| 98 | 12/4/2018 | 229 | 90% | 1091.5 |
| 99 | 12/5/2018 | 230 | 88% | 1043.4 |
| 100 | 12/5/2018 | 230 | 91% | 895.4 |
| 101 | 12/6/2018 | 231 | 89% | 1039.7 |
| 102 | 12/10/2018 | 235 | 88% | 910.2 |
| 103 | 12/11/2018 | 236 | 91% | 1069.3 |
| 104 | 12/14/2018 | 239 | 91% | 1058.2 |
| 105 | 12/14/2018 | 239 | 91% | 906.5 |
| 106 | 12/18/2018 | 243 | 89% | 1006.4 |
| 107 | 1/2/2019 | 258 | 93% | 984.2 |
| 108 | 1/4/2019 | 260 | 89% | 984.2 |
| 109 | 1/7/2019 | 263 | 89% | 847.3 |
| 110 | 1/8/2019 | 264 | 88% | 939.8 |
| 111 | 1/9/2019 | 265 | 90% | 958.3 |
| 112 | 1/10/2019 | 266 | 88% | 943.5 |
| 113 | 1/10/2019 | 266 | 89% | 906.5 |
| 114 | 1/11/2019 | 267 | 85% | 906.5 |
| 115 | 1/14/2019 | 270 | 86% | 836.2 |
| 116 | 1/15/2019 | 271 | 87% | 902.8 |
| 117 | 1/17/2019 | 273 | 89% | 710.4 |
| 118 | 1/18/2019 | 274 | 85% | 880.6 |
| 119 | 1/22/2019 | 278 | 88% | 873.2 |
| 120 | 1/23/2019 | 279 | 89% | 928.7 |
| 121 | 1/24/2019 | 280 | 87% | 921.3 |
| 122 | 1/25/2019 | 281 | 86% | 906.5 |
| 123 | 1/28/2019 | 284 | 88% | 858.4 |
| 124 | 1/30/2019 | 286 | 90% | 910.2 |
| 125 | 2/1/2019 | 288 | 89% | 906.5 |
| 126 | 2/4/2019 | 291 | 89% | 862.1 |
| 127 | 2/5/2019 | 292 | 87% | 891.7 |
| 128 | 2/6/2019 | 293 | 88% | 888 |
| 129 | 2/7/2019 | 294 | 87% | 899.1 |
| 130 | 2/11/2019 | 298 | 86% | 784.4 |
| 131 | 2/13/2019 | 300 | 87% | 865.8 |
| 132 | 2/14/2019 | 301 | 88% | 876.9 |
| 133 | 2/15/2019 | 302 | 88% | 851 |
| 134 | 2/20/2019 | 307 | 89% | 873.2 |
| 135 | 2/21/2019 | 308 | 88% | 854.7 |
| 136 | 2/22/2019 | 309 | 88% | 862.1 |
| 137 | 2/22/2019 | 309 | 89% | 791.8 |
| 138 | 2/25/2019 | 312 | 86% | 799.2 |
| 139 | 2/27/2019 | 314 | 90% | 902.8 |
| 140 | 2/28/2019 | 315 | 91% | 765.9 |
| 141 | 3/4/2019 | 319 | 90% | 817.7 |
| 142 | 3/5/2019 | 320 | 88% | 847.3 |
| 143 | 3/6/2019 | 321 | 91% | 880.6 |
| 144 | 3/7/2019 | 322 | 88% | 858.4 |
| 145 | 3/8/2019 | 323 | 90% | 869.5 |
| 146 | 3/14/2019 | 329 | 89% | 851 |
| 147 | 3/18/2019 | 333 | 90% | 777 |
| 148 | 3/21/2019 | 336 | 92% | 828.8 |
| 149 | 3/22/2019 | 337 | 87% | 788.1 |
| 150 | 3/25/2019 | 340 | 84% | 715.21 |
| 151 | 3/26/2019 | 341 | 88% | 821.4 |
| 152 | 3/29/2019 | 344 | 89% | 780.7 |
| 153 | 4/1/2019 | 347 | 88% | 758.5 |
| 154 | 4/2/2019 | 348 | 89% | 784.4 |
| 155 | 4/4/2019 | 350 | 87% | 777 |
| 156 | 4/5/2019 | 351 | 87% | 762.2 |
| 157 | 4/9/2019 | 355 | 86% | 706.7 |
| 158 | 4/10/2019 | 356 | 88% | 758.5 |
| 159 | 4/11/2019 | 357 | 88% | 680.8 |
| 160 | 4/16/2019 | 362 | 86% | 740 |
| 161 | 4/17/2019 | 363 | 87% | 754.8 |
| 162 | 4/19/2019 | 365 | 89% | 773.3 |
| 163 | 4/22/2019 | 368 | 88% | 695.6 |
| 164 | 4/23/2019 | 369 | 88% | 747.4 |
| 165 | 4/24/2019 | 370 | 87% | 736.3 |
| 166 | 4/24/2019 | 370 | 89% | 669.7 |
| 167 | 4/25/2019 | 371 | 88% | 754.8 |
| 168 | 4/25/2019 | 371 | 86% | 703.37 |
| 169 | 4/26/2019 | 372 | 90% | 762.2 |
| 170 | 4/29/2019 | 375 | 89% | 743.7 |
| 171 | 4/30/2019 | 376 | 88% | 740 |
| 172 | 5/1/2019 | 377 | 86% | 714.1 |
| 173 | 5/2/2019 | 378 | 89% | 743.7 |
| 174 | 5/2/2019 | 378 | 88% | 658.6 |
| 175 | 5/7/2019 | 383 | 85% | 625.3 |

Table SM4. Drug product release criteria for [^68^Ga]Ga-PSMA-11

| Test | Acceptance Criteria | Results |
| --- | --- | --- |
| Appearance | Clear, colorless solution. free from particulate matter | conforms |
| Radionuclidic identity | 62-74 min | 66-70 min |
| Radionuclidic purity | 511 ± 51 keV | conforms |
| Radiochemical identity | R_T_ of [^68^Ga]Ga-PSMA-11should be ± 10% the R_T_ of Ga-PSMA-11 std | RT std: 7.16 min  RT FDP: 7.37 min  Δ RT: 2.93% |
| Radiochemical purity | ≥ 96% | > 99% |
| Chemical purity | ≥ 90% | > 99% |
| ^68^Ge impurity | < 0.001% | < 0.0000069% |
| pH | 4.5-8.0 | 5.0-5.5 |
| Bubble Point | ≥ 50 psi | 52-58 psi |
| LaL (pyrogenicity) test | <29 EU/mL | <5.00 EU/mL |
| sterility | Pass (no microbial growth) | pass |

Figure SM5. Typical QC Chromatogram for [^68^Ga]Ga-PSMA-11.


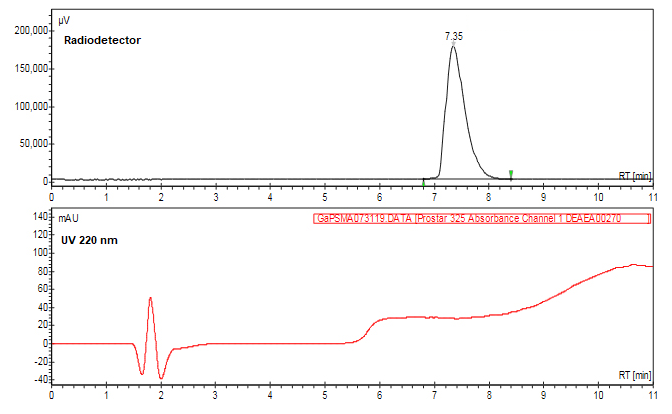

Supplement: Supplementary file 1 — Additional file 1: Table S1. Data compilation: 68Ga elution yields and 68Ge contents. Table S2. Results from ICP-MS for metal contamination. Table S3. Compilation of [68Ga]Ga-PSMA-11 Syntheses. Table S4. Drug product release criteria for [68Ga]Ga-PSMA-11. Figure S5. Typical QC Chromatogram for [68Ga]Ga-PSMA-11. [file 41181_2019_87_MOESM1_ESM.docx]
